# Supplementary material for: Establishment of methanogen bacterial interactions during the preweaning period of dairy cattle
Source: PLoS One. 2024 Sep 20;19(9):e0310648. doi: 10.1371/journal.pone.0310648 (PMC11414971; doi:10.1371/journal.pone.0310648)
Supplement: S1 Table — (DOCX) [file pone.0310648.s001.docx]

**S1 Table**

| **Calf age**  **(weeks)** | **Volume of acidified milk given**  **per calf 2x/day** | **Amount of starter grain given**  **per calf 1x/day** |
| --- | --- | --- |
| 1 | 2 quarts | 0 lbs |
|  |  | 2 lbs |
| 2 | 3 quarts | 2 lbs |
| 3 | 4 quarts | 2.5 lbs |
| 4 | 5 quarts | 3 lbs |
| 5 | 4 quarts | 3.5 lbs |
| 6 | 3 quarts | 4 lbs |
| 7 | 2 quarts | 4.5 lbs |
| 8 | Weaning | 5 lbs |
| **Composition of starter grain** | | |
| Monensin | 72 g/ton | |
| Crude protein | Minimum 18.0% | |
| Crude fat | Minimum 4.25% | |
| Crude fiber | Maximum 8.0% | |
| Ingredients | Grain products, plant protein products, processed grain,  byproducts, molasses, vegetable oil, calcium carbonate,  blood meal, salt, vitamin E supplement, mineral oil,  premium yeast fraction, sodium selenite,  vitamin A acetate/D3 supplement, copper sulfate,  magnesium oxide, magnesium sulfate, potassium sulfate,  zinc sulfated, ethylenediamine, dihydroidide, cobalt carbonate | |
