# Supplementary material for: Establishment of methanogen bacterial interactions during the preweaning period of dairy cattle
Source: PLoS One. 2024 Sep 20;19(9):e0310648. doi: 10.1371/journal.pone.0310648 (PMC11414971; doi:10.1371/journal.pone.0310648)
Supplement: Attachment — Submitted filename: Response to reviewers.docx [file pone.0310648.s003.docx]

**Editors’ comments**

**1. Please ensure that your manuscript meets PLOS ONE's style requirements, including those for file naming. The PLOS ONE style templates can be found at**

**[https://journals.plos.org/plosone/s/file?id=wjVg/PLOSOne_formatting_sample_main_body.pdf](https://urldefense.com/v3/__https:/journals.plos.org/plosone/s/file?id=wjVg*PLOSOne_formatting_sample_main_body.pdf__;Lw!!IBzWLUs!VqOecGBkus3ph_0-iwXiQdEHBFDE16k3bFTeX14BzfAAprzfFxrwHz5OY6FU0TFVm9LfojM1XiVEQFPVZMt_EY8p$) and** [**https://journals.plos.org/plosone/s/file?id=ba62/PLOSOne_formatting_sample_title_authors_affiliations.pdf**](https://urldefense.com/v3/__https:/journals.plos.org/plosone/s/file?id=ba62*PLOSOne_formatting_sample_title_authors_affiliations.pdf__;Lw!!IBzWLUs!VqOecGBkus3ph_0-iwXiQdEHBFDE16k3bFTeX14BzfAAprzfFxrwHz5OY6FU0TFVm9LfojM1XiVEQFPVZNsSXguK$)

***AU:*** *We have revised the manuscript to adhere to PLOS ONE's style requirements. This includes updates to figure citations, file naming conventions, and references to align with the guidelines provided.*

**2. Thank you for stating the following financial disclosure:**

**“This research was funded by the United States Department of Agriculture National Institute of Food and Agriculture (Grant #PENV570262;** [**https://nifa.usda.gov/us-states-and-territories/pennsylvania)**](https://nifa.usda.gov/us-states-and-territories/pennsylvania))**.”**

**Please state what role the funders took in the study.  If the funders had no role, please state: "The funders had no role in study design, data collection and analysis, decision to publish, or preparation of the manuscript." If this statement is not correct you must amend it as needed. Please include this amended Role of Funder statement in your cover letter; we will change the online submission form on your behalf.**

***AU:*** *As requested, we have included the Role of Funder statement in the cover letter.*

**3. Please update your submission to use the PLOS LaTeX template. The template and more information on our requirements for LaTeX submissions can be found at** [**http://journals.plos.org/plosone/s/latex**](https://urldefense.com/v3/__http:/journals.plos.org/plosone/s/latex__;!!IBzWLUs!VqOecGBkus3ph_0-iwXiQdEHBFDE16k3bFTeX14BzfAAprzfFxrwHz5OY6FU0TFVm9LfojM1XiVEQFPVZOupOAzZ$)**.**

***AU:*** *We have updated our manuscript according to PLOS submission guidelines.*

**4. Please review your reference list to ensure that it is complete and correct. If you have cited papers that have been retracted, please include the rationale for doing so in the manuscript text, or remove these references and replace them with relevant current references. Any changes to the reference list should be mentioned in the rebuttal letter that accompanies your revised manuscript. If you need to cite a retracted article, indicate the article’s retracted status in the References list and also include a citation and full reference for the retraction notice.**

***AU:*** *We have reviewed and updated references and citations*

**5. Review Comments to the Author**

**Please use the space provided to explain your answers to the questions above. You may also include additional comments for the author, including concerns about dual publication, research ethics, or publication ethics. (Please upload your review as an attachment if it exceeds 20,000 characters)**

**Reviewer #1: Overall, this is a beautiful study revealing microbial dynamics in developing animals during the preweaning period of dairy cattle. This is crucial for understanding and optimizing rumen development, which significantly impacts the future productivity and health of the animals.**

*Thank you for your comments. We have sought to address the specific critiques below.*

**Comments**

**Line 84-88: Please rewrite the objective and your hypothesis to succinctly conform with the abstract. There is a disjoint in the sentence especially line 88……. which further dictates….. Sentences like this should not stand here.**

***AU:*** *We have revised the objective and hypothesis for clarity. The sentence has been improved as suggested (Lines 84-94).*

**Line 98: Remove ad libtum from this sentence and leave as…… grain consumption was provided………**

***AU:*** *We have removed the term "ad libitum" as suggested.*

**Line 99: How did you collect your fecal samples? Was the feces collected via stimulation or was picked from the floor……. It would be important to have a line of sentence describing this.**

***AU:*** *We collected fecal samples via rectal stimulation. This information has been added to the manuscript (Lines 107-110).*

**Line 220: Replace accompaniment with follow up study…….**

***AU:*** *Agreed and corrected.*

**Line 144: Rarefied or rarified… Typo error, this should be Rarefied.**

***AU:*** *Thank you. We have corrected it to "Rarefied."*

**Reviewer #2: The manuscript presents a valuable contribution to understanding methanogen colonization in neonatal dairy calves and its potential impact on methane emissions. The study is methodologically sound and well-referenced, providing a comprehensive analysis of the temporal dynamics of methanogenic archaea and their interactions with bacteria. However, some information about Materials and methods are missing, likewise some improvements need to be made.**

Thank you for your comments. We have sought to address the specific critiques below.

**Abstract:**

**Include age and average weight of the calves at the point of the study**

***AU:*** *The age of the calves and average birth weight is included in the abstract as suggested (Lines 32-35).*

**Introduction:**

**Add references at line 56, and 58.**

***AU:*** *We have revised the sentences as requested and added two references: Johnson & Johnson (1995) and Wolin (1981).*

**All acronyms should be defined.**

**While some of the target audiences are likely familiar with technical terms, ensuring that complex terminology (e.g., "methylotrophic methanogens", "hydrogenotrophic methanogens") is clearly defined upon first use can enhance accessibility.**

***AU:*** *We have defined both "methylotrophic methanogens", and "hydrogenotrophic methanogens (Lines 74-77)*

**The knowledge gap mentioned in lines 81-83 is critical but could be more explicitly stated. A clear statement of what is known versus what is not would sharpen the focus.**

***AU:*** *We have revised the text to clearly state the knowledge gap (Lines 84-88).*

**Materials and Methods:**

**Include the average weight of the calves at the point of the study**

***AU:*** *We included the average birth weight of the calves (lines 102-103).*

**Why wasn’t a control group (group of calves not receiving the same diet) included in the study to help provide comparative insights?**

***AU:*** *The primary objective of this study was to understand the temporal patterns of methanogenic archaea, rather than to compare different diets. As such, a control group was not included. All calves followed the same dietary protocol: they received acidified milk until 8 weeks of age and were then switched to a diet of grain and hay, as per the standard feeding schedule on our dairy farm. As such there is no treatment but we compared methanogenic communities by age of calves.*

**Provide detailed information about the acidified milk and the grain and hay diet content (Diet Table).**

***AU:*** *We have now included a detailed Diet Table as Supplementary Table 1 in the manuscript, which provides information on the composition of the acidified milk, grain, and hay diets.*

**Why were fecal samples collected only and rumen fluid samples were not collected to give more information about the ruminal content and the microbial populations of the calves, similar to the study by Friedman et al., 2017.**

***AU:*** *Rumen is not fully developed, and calves are considered monogastric until weaning. According to Friedman et al paper, samples were collected at Wk2 and then 2 months which is not similar to our study. Our goal was to follow temporal dynamics and sampling for rumen contents at that frequency will be considered invasive sampling. Collecting rumen at birth through 4-6 weeks of age is also considered invasive and may not represent true composition of rumen microbiota as rumen is still developing. Therefore, we relied on fecal sampling.*

**Details of fecal collection procedure should be included**

***AU:*** *The fecal collection procedure is now described in (lines 107-110).*

**What time during the day were the fecal samples collected from each calf, was it after or before meals?**

***AU:*** *We typically collect fecal samples 2h post-feeding, this information has been updated in text (Line 108).*

**Results**

**Line 138: Were these four experimental samples removed from other analyses?**

***AU:*** *Yes, we removed the four experimental samples from all microbiome analyses.*

**Line 179 needs to be improved (Methanogenic)**

***AU:*** *We have replaced “Methanogenic” with “Archaea”.*

**Line 226 shows should be show**

***AU:*** *We have replaced “shows” with “show”.*

**Were residuals checked for normality, and how were potential outliers handled?**

***AU:***

*We used generalized estimating equations (GEE) statistical model to analyze alpha diversity and for taxonomy. In GEE, the primary focus is on the population-averaged effects rather than individual-level predictions, and as such, the normality of residuals is not a strict requirement. GEE is robust to misspecifications of the working correlation structure, and the estimates of the regression coefficients are consistent even if the residuals are not normally distributed. Therefore, we did not check residuals for normality, as it is not a prerequisite for the validity of GEE results.*

*In our analysis, we used boxplots and interquartile range (IQR) statistics to identify potential outliers and found two animals at Week 2 that were outliers. We then ran the GEE model without these two observations. The results were consistent whether or not we included the outliers, indicating that they did not significantly impact our findings.*

**Discussion**

**The transition between discussing bacterial and methanogen communities seems a bit abrupt, improving the flow would help readability**

***AU:*** *Thank you, we have revised the sentence as suggested (Lines 268-281).*

**Explain why acidified milk was chosen and how it might influence the microbial dynamics compared to other feeding strategies**

***AU:*** *We have added justification for using acidified milk (Lines 282-300).*

**Rephrase line 232 to “Surprisingly, the correlation analysis revealed that, at any given time point, only one of the two genera was present, and they did not co-occur.” to improve clarity.**

***AU:*** *We have rephrased the sentence as suggested (Lines 249-264).*

**Simplifying the language where possible and defining key terms would make the discussion more accessible.**

**The use of terms like "flux" and "clusters" needs to be clearly defined in the context of microbial community dynamics to avoid ambiguity.**

***AU:*** *flux eliminated, grouping patterns added in parenthesis for clusters*

**Lines 263-266: The mention of similar interactions in mature rumen and low-methane yield phenotype cows is interesting but needs more context and explanation. How do these findings compare with other studies on methanogen-bacteria interactions?**

***AU:*** *We have revised the sentences as suggested (Lines 325-333)*

**Create a sub-heading for the Conclusion**

***AU:*** *A separate sub-heading has been created for the Conclusion.*
